# Resolutions Adopted by the New-York State Medical Society, at Its Last Annual Meeting (February,) Relative to Medical Reform in This State

**Published:** 1846-06-01

**Authors:** 


*Resolutions adopted by the New-York State Medical Society, at its last annual meeting (February,) relative to medical reform in this State.*

We insert the following resolutions of the State Medical Society, and we would express the hope that each County Society in the State will take the subjects ino deliberate consideration, and not fail to respond to the call to report upon them in season, for the action of the State Society at its next annual meeting. We shall offer our views on the resolutions in a future number. These resolutions are all that we find for quotation or comment in the proceedings of the Society. We shall not, therefore, bestow farther review upon them.—*Ed. Buff. Jour.*

1st. *Resolved,* That every candidate for license to practice physic and surgery, should be required to give evidence to the board, by whom he is examined, that he has received a good academical education, and that he has studied physic and surgery not less than four years with some regularly licensed physician. That he possesses a competent knowledge of physic and surgery; that he has personally assisted in the dissection of at least two good anatomical subjects, under the direction of a regularly educated physician, or in some incorporated medical college in this state; that he is practically acquainted with chemistry and botany, and has presented the board with an essay on some medical subject, written by himself; and that he is twenty-one years of age, and of good moral character.

2d. *Resolved,* That a board of censors, to consist of not more than five from each of the censorial districts, be chosen—the choice to be made from the candidates who may be nominated by the county medical societies, composing the respective censorial districts—by a majority of the votes of all the members of the State Medical Society present and voting, at any annual meeting thereof. The term of office of each censor, in the respective censorial district, to be determined at the first election by lot, and thereafter the vacancy of one in each censorial district, to be filled by the State Society at its annual meeting; seven of said board of censors to constitute a quorum for the transaction of business—to meet at such times aud places, and be governed by such rules and regulations, and receive such compensation for their services, as the State Medical Society shall, from time to time, direct. The board of censors thus constituted, shall examine all candidates for license to practice physic and surgery; and to such as are found qualified, agreeably to the requirements of the preceding resolution, it shall be their duty to grant a license to practice physic and surgery, under the auspices of this society, after filing their certificates, as now required by law. Said board may also examine such candidates for the degree of Doctor of Medicine, as have been practitioners of medicine in good standing, for at least five years, and by a vote of two-thirds of the board of examiners present, may recommend such candidates, to the Regents of the University, as suitable persons on whom to confer said degree. Each person licensed, to pay to the president of the board of censors, for the use of this Society, twenty-five dollars; and each person who is recommended as entitled to the degree of Doctor of Medicine, to pay as aforesaid, the sum of thirty-five dollars, and no license to be granted, or degree conferred, until said fees are paid. And that said board annually report their doings to this body. And all laws relating to granting licenses to practice physic and surgery, and to conferring the degree of Doctor of Medicine in this State, inconsistent with the above regulations, to be repealed.

3d. *Resolved*, That this Society should appoint eight committees, corresponding with the number of senatorial districts in the State, whose duty it shall be to carefully investigate such subjects as shall be referred to them, and report annually, in writing, the result of their investigations, to the regular meetings of this Society; and that, as far as possible, the necessary expenses of such investigations be audited and paid out of the treasury of this Society.

4. *Resolved*, That in order successfully to raise the standard of medical education, it is necessary, at the same time, to improve primary popular education, in reference to the subject of medicine; that in order to appreciate knowledge, it is necessary to understand something of the principles upon which it is based; and that physiology, chemistry and botany should constitute essential parts of a common school education.

The following was then adopted:

*Resolved*, That the resolutions in reference to medical education, as revised and adopted at this meeting, be committed to a committee of three, whose duty it shall be to lay a copy of the same before the officers of each county medical society and college in the State, asking definite and direct action thereon, by the societies and colleges represented by them, and that the committee report the result of such action at the next annual meeting of this Society. And that N. S. Davis, T. W. Blatchford, and J. A. Wing, be said committee.

